# Sea Urchin Gonad Enhancement and Coloration: Nutritional Strategies and Ecological Considerations

**DOI:** 10.3390/ani15243583

**Published:** 2025-12-12

**Authors:** Jeremie Bauer, Jorge Olmos

**Affiliations:** Marine Biotechnology Department, Centro de Investigación Científica y de Educación Superior de Ensenada (CICESE), Ensenada 22860, Baja California, Mexico; jbauer@cicese.mx

**Keywords:** echinoderm aquaculture, uni production, functional feed, urchin feed formulation, restorative aquaculture, sea urchin barrens

## Abstract

This review analyzes current research on short-term culture of sea urchin from barrens through formulated feed, addressing the need for sustainable aquaculture practices and ecological restoration of kelp forests. We compare the results of multiple studies to identify the optimal feed composition to induce gonad growth and coloration. Our analysis suggests that macroalgae are the best feed ingredients to improve gonad growth and coloration; however, environmental and economic challenges persist in expanding sea urchin production with these types of ingredients. Plant-based protein sources like soy have emerged as a potential cost-effective alternative to fish products; nevertheless, the presence of antinutritional factors in soy products limits their inclusion in formulated feed. Regarding the composition and amount of lipids, we found that they are critical macronutrients in gonad development. The review also explores the potential of sea urchin aquaculture in mitigating urchin barrens and restoring kelp forests, highlighting the interplay between ecological and economic factors. We identify key knowledge gaps and propose future research directions, including large-scale economic viability assessments, novel feed additives, and integrated multitrophic aquaculture systems. These findings have significant implications for developing sustainable and economically viable sea urchin aquaculture, potentially transforming urchin barrens into productive ecosystems while meeting market demand for roe.

## Simple Summary

Sea urchins are valued for their edible reproductive organs, called gonads or roe, which are considered a delicacy in many cultures. However, in some areas, overpopulation of sea urchins has led to overgrazing of kelp forests, creating underwater deserts called urchin barrens. While sea urchins have great commercial value, those from barrens yield poor-quality gonads due to nutritional deficiencies, rendering them commercially unviable. This review examines how short-term sea urchin ranching using specially designed feeds could generate commercially viable gonads. We analyzed research on different feed ingredients to determine what helps sea urchins grow larger, better-colored gonads. We found that seaweed-based feeds work best but are expensive and environmentally challenging to obtain in large quantities with standard quality. Plant-based proteins like soybean meal show promise as more sustainable and affordable alternatives. The right amount of fats in the diet is also critical for gonad development. Farming sea urchins from barren areas could help restore kelp forests while producing valuable seafood, but more research is needed on developing economically viable and sustainable formulated feeds with new supplements.

## 1. Introduction

**Figure 1:**
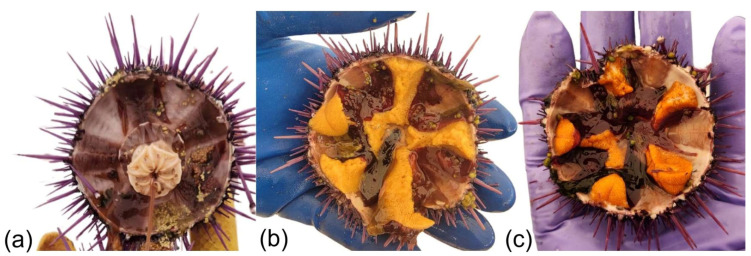
Purple sea urchin, *Strongylocentrotus purpuratus*, gonads. (**a**) Sea urchin collected from urchin barren with no marketable gonads; (**b**) yellow and (**c**) orange gonads. All images constitute original content from the authors and are used as examples for coloration.

The global market for sea urchin gonads, known as uni or roe, has experienced substantial growth driven by expanding consumer demand and high market value [1]. Sea urchin gonads are preferred for their large size [2], bright orange or yellow color [3] (Figure 1b,c), high content of sweet-tasting alanine, strong umami, and low content of bitter-tasting arginine [4]. In addition, Takagi et al. [4] show the role of urchin diet in relation to odor-active compounds from butyl acetate and amino acids for umami as essential flavor components of high-quality gonads. The annual average price of sea urchin gonads at the Tokyo Metropolitan Central Wholesale Market, the largest sea urchin market in the world, has nearly doubled over the past two decades, with uni reaching USD 131/kg in 2018 [5]. However, the growing demand for uni has resulted in significant pressure on wild sea urchin populations (e.g., in the Mediterranean region), leading to the depletion of natural stocks and disrupting marine ecosystem balance [6,7,8].

Paradoxically, in other coastal areas, climate change-related processes combined with the overharvesting or disappearance of sea urchin predators have led to the proliferation of sea urchin populations, creating “urchin barrens”—areas of low biodiversity dominated by sea urchins that have overgrazed macroalgae [9,10,11,12,13]. In these barrens, urchin densities can reach hundreds of individuals m^−2^ in three-dimensional aggregations that affect kilometers of coastline [14]. While sea urchins have great commercial value, those from barrens yield poor-quality gonads or even no gonads at all (Figure 1a) due to nutritional deficiencies, rendering them commercially unviable [15,16,17,18,19,20].

The expansion of urchin barrens over the past five decades has been causing significant ecological consequences, particularly in temperate rocky ecosystems dominated by kelp forests [4,10,13,21,22,23,24,25,26,27]. These macroalgal forests, primarily comprising brown algae of the order Laminariales, red algae, and green algae, are among the most productive marine biomes [28,29,30]. However, kelp forests have experienced an alarming 40–60% decline worldwide due to complex abiotic and biotic stressors, with many regions transitioning to urchin barrens [22,30].

The transition from kelp forests to urchin barrens has been documented globally, including areas in the U.S.A, Mexico, Australia, Tasmania, New Zealand, Korea, Japan, and Norway [21,22,23,27,31,32,33,34,35,36,37,38]. Once established, urchin barrens can persist for years or even decades due to positive feedback mechanisms, making the transition back to kelp forests difficult [39]. One short-term strategy to restore kelp forests is to significantly reduce urchin abundances through removal efforts [21]. Nonetheless, the scalability and economic feasibility of such removals require innovative biotechnological solutions to add value to sea urchins obtained from barrens.

Aquaculture represents an opportunity to aid in the removal of sea urchins from barrens and simultaneously address market demand for high-quality uni. While full life-cycle aquaculture exists, its profitability has been hindered by the relatively slow growth of sea urchins, taking up to 3 years to reach market size [4,40,41,42]. In contrast, gonad enhancement of wild-caught sea urchins can be achieved in just 6–12 weeks [17,18,20,43]. The latter approach, known as sea urchin ranching, has been proposed as a restorative aquaculture strategy to generate economic benefits for coastal communities and induce the production of sustainable food while restoring macroalgae in local removal areas [7,44,45].

For example, the Norwegian coastline has experienced extensive kelp forest decline due to green sea urchin (*Strongylocentrotus droebachiensis*) overpopulation, with urchin barrens covering hundreds of kilometers of formerly productive kelp habitat. In response, Norway has pioneered both mechanical removal programs and experimental quicklime (CaO) treatments to reduce urchin densities and facilitate macroalgal recovery [35]. Within 2 years post-removal, macroalgal cover increased from 0% to 40–80% at depths of 2 and 5 m, respectively. As pioneers, Norwegian sea urchin ranching operations have successfully enhanced wild-caught *S. droebachiensis* to commercial quality within 9 weeks using formulated feeds [46]. This case study indicates the potential of sea urchin ranching as an integrative strategy to increase the economic and long-term viability of kelp forest restoration programs.

Today, sea urchin ranching operations remain limited but are growing, with pilot projects and small-scale commercial companies operating in Japan, Norway, Canada, New Zealand, and the United States [7]. Feed is the most important factor in determining the success of sea urchin ranching. Macroalgae are the natural food of sea urchins; however high prices, unstable supply, temporal and seasonal variation in quality and/or quantity, negative environmental effects from large-scale harvesting of wild populations, and the scarcity in affected barren areas make macroalgae unlikely to be commercially viable for scaling up operations [47,48,49,50]. Therefore, formulated feed is crucial for the ongoing expansion and sustainable development of this industry.

Formulated feed has the advantage of balanced and precise nutrition, convenient storage, stable supply, and effectiveness in providing functional substances and bioactive compounds. Additionally, formulated feed can promote higher gonad indices in sea urchins with better conversion ratios than macroalgae [17,51,52]. In comparison, in the South African abalone (a gastropod herbivore from the genus *Haliotis*) industry, the use of formulated feed is generally less expensive than the use of fresh macroalgae due to availability, accessibility, transport, and food conversion ratios [53]. In that sense, one of the most pressing goals in sea urchin aquaculture is the development of formulated feed that can effectively mimic the nutritional complexity and variability of natural diets while maintaining palatability, consumability and digestibility [7,54]. The challenge extends beyond simply matching the macronutrient profiles of macroalgae, but also involves the intricate balance of pigments and other bioactive compounds to achieve gonad enhancement and quality while simultaneously ensuring the cost-effectiveness and sustainability of the feed [50,55,56].

Despite the progress in formulated feed development, challenges persist in optimizing nutrient utilization and mitigating the potential negative effects of plant-based ingredients. Biotechnological approaches, particularly the use of functional feed additives such as probiotics, prebiotics, phytobiotics, and enzymes, offer promising solutions to these challenges [57]. Probiotics (e.g., *Bacillus* spp.) can enhance nutrient utilization, improve growth performance, strengthen immune response, and enhance reproductive performance in aquatic species; moreover, probiotics can improve water quality and reduce the use of antibiotics in aquatic systems [58,59,60,61,62,63]. Prebiotics (e.g., inulin, fructooligosaccharides and transgalactooligosaccharides) may improve gut health and feed efficiency [64]. Phytobiotics (e.g., alkaloids, terpenoids, tannins, saponins, glycosides, flavonoids, phenolics, steroids or essential oils), derived from plants, have shown potential in reducing oxidative stress, increasing antimicrobial activity, and enhancing growth performance [65]. Enzymes (e.g., phytase, protease, carbohydrase, and lipase), particularly those targeting antinutritional factors in plant-based ingredients, can significantly improve nutrient availability and reduce the environmental impacts of aquaculture [66]. These biotechnological interventions may play a crucial role in developing more efficient and sustainable feed formulations for sea urchin ranching, potentially addressing both nutritional and ecological concerns.

The economic imperative for proper coloration becomes particularly acute in the context of sea urchin ranching from barren areas, where urchins typically present with pale, commercially unviable gonads due to nutritional deficiencies. Without feed formulations capable of reliably producing market-preferred colors within the short culture period (6–12 weeks) necessary for economic feasibility, the restorative aquaculture model cannot succeed commercially. Thus, understanding the nutritional factors—particularly the carotenoid content, composition, and lipids necessary for their absorption—that determine gonad coloration is as critical as optimizing for gonad size and growth rate.

The objective of this review is to analyze the effects of formulated feed on sea urchin gonad enhancement and coloration across multiple sea urchin barren-forming species. By synthesizing current research, we aim to evaluate the sustainability of feed composition and its impact on gonadosomatic index growth rate and marketable coloration qualities. This comprehensive analysis will provide insights into the nutritional strategies, economic and ecological considerations, and biotechnological frontiers of short-term sea urchin culture, with implications for both the aquaculture industry and marine ecosystem management.

## 2. Sea Urchin Species with Ranching Potential

The varied ecological contexts and several sea urchin species that form barrens demand a diverse approach to sea urchin ranching. Each species has unique characteristics that influence their suitability for ranching operations (Table 1), underscoring the need for species-specific strategies in feed formulation.

The most extensively ranched species is the green sea urchin *S. droebachiensis*. Its prominence is largely due to decades of research and development in Norway, which has established a strong foundation for commercial ranching practices [46,49,67,68,69,70,71]. Moreover, *S. droebachiensis* possesses several characteristics that make it particularly suitable for ranching operations (Table 1): (1) It has a wide temperature tolerance range with an Arctic boreal distribution that facilitates ranching initiatives across multiple regions [70]; (2) it forms extensive barrens, causing significant ecological impacts that ranching can help mitigate [72]; (3) it exhibits rapid gonad development under optimal feeding conditions [52]; and (4) its roe generally meets good market quality standards [49].

Another species with outstanding potential is the purple sea urchin, *Strongylocentrotus purpuratus*. Its wide distribution along the North American Pacific coast, temperature range (12–20 °C), and rapid gonad growth (8 weeks) under optimal feed condition make it an attractive option for ranching (Table 1). Additionally, it forms extensive barrens, resulting in a >90% decline in kelp forests in some areas [22], impacting valuable fisheries like the red sea urchin *Mesocentrotus franciscanus*, which is worrisome for small-scale fishing communities that depend on these resources [73,74]. Moreover, the full-year open fishery for *S. purpuratus* in Baja California, Mexico [75], provides a critical advantage for continuous ranching operations, enhancing the economic viability and scalability of such initiatives in the region.

Other sea urchin species that show promise for ranching are shown in Table 1. These include *M. franciscanus* [76], *Evechinus chloroticus* [77,78], *Tripneustes gratilla* [47,79], *Heliocidaris erythrogramma* [16,56,80,81,82], *Paracentrotus lividus* [83,84,85], and *Mesocentrotus nudus* [4,5,6,18,20,86,87]. It is important to note that different sea urchin species may respond differently to formulated feed in terms of gonad enhancement and quality. Therefore, the results and interpretations presented in this review should be approached with caution, and further species-specific research is warranted to optimize feed formulation for each target species in sea urchin ranching operations.

**Table 1:** Characteristics of sea urchin species with potential for commercial ranching and gonad enhancement.

Species	Temperature Range (°C)	Key Distribution Areas	Wild Densities (ind/m^2^)	Max Size (mm)	GI (%)	Market Demand	ERP	Key Considerations for Ranching	References
*Strongylocentrotus droebachiensis*	−1–20	Arctic boreal distribution	0.1–70 (barrens up to 300)	80	21 with FF in 9 weeks.	High	High	Adapts well to various feed types; potential for kelp forest restoration.	[32,52,71,88]
*Strongylocentrotus purpuratus*	12–20	Northeastern Pacific from Alaska, U.S.A. to Isla Cedros, Baja California, Mexico	0.5–50 (barrens up to 200)	100	24 with FF vs. 11.7 with kelp *Macrocystis pyrifera* in 9 weeks.	High	High	Forms extensive barrens; good candidate for restorative aquaculture.	[17,22,89,90,91,92,93]
*Mesocentrotus nudus* (formerly *Strongylocentrotus nudus*)	5–25	East Asia (China, Russia, Korea, Japan) Range extension towards the Pacific coast of Japan-Akkeshi Bay)	0.5–10 (barrens up to 100)	80	8.9 to 24 in 6 weeks fed sporophylls of *Undaria pinnatifida* 9.1 to 23.2 with formulated feed. 16 with *Saccharina ochotensis*. Up to 38.5 in an *Eisenia bicyclis* bed and 14.7 in barrens.	Highest prices	High	Valuable species in Asian markets; responsive to both formulated and macroalgae diets.	[5,20,48,50,86,94,95,96]
*Mesocentrotus franciscanus* (formerly *Strongylocentrotus franciscanus*)	8–14	Northeastern Pacific from Alaska to Baja California	0.1–30 (barrens up to 40)	180	20 with FF and kelp *Nereocystis luetkaena* in 12 weeks vs. 11 from the wild organisms from the fishery in the same season.	High	Medium	Valuable fishery species; bigger gonads but slower growth rates than *S. purpuratus*.	[76,90,91,93,97,98,99,100,101]
*Evechinus chloroticus*	10–18	Aotearoa New Zealand	0.1–20 (barrens up to 50)	170	12.3 with FF in 8 weeks.	Medium	Medium	Important in New Zealand fisheries; requires specific feed formulations.	[77,102,103,104]
*Tripneustes gratilla*	24–30	Indo-Pacific with ranching in South Africa	0.1–10	150	22 with FF and 12 with green *Ulva rigida* in 12 weeks.	Medium	Low	Fast growth; potential for tropical aquaculture.	[47,79,105,106,107]
*Heliocidaris erythrogramma*	12–23	Southern Australia	1–4 (barrens 10–192)	100	21.7 with FF in 12 weeks.	Medium	Medium	Adapted to temperate waters; good response to formulated feed.	[31,82,108,109,110,111]
*Paracentrotus lividus*	10–25	Northeastern Atlantic and Mediterranean.	1–50 (barrens > 15)	70	19 with FF in 17 weeks.	High	Medium	Important in European markets; sensitive to handling.	[84,112,113,114,115,116]

Note: population densities and maximum gonadosomatic indices (GIs) can vary significantly depending on geographical location, season, and environmental conditions. The values presented here are representative ranges based on the available literature but may not encompass all possible scenarios. Gonad enhancement results are from controlled studies and may differ in commercial settings. Feed responses may vary based on specific formulations and environmental factors. Market demand and ecological restoration potential are general assessments and may change over time or differ by region. Readers are encouraged to consult the most recent and location-specific literature for precise figures and up-to-date information. ERP = ecological restoration potential; FF = formulated feed.

## 3. Sea Urchin Formulated Feed Development

### 3.1. Sea Urchin Natural Diets

The natural diet of sea urchins, primarily composed of various macroalgae species, plays a crucial role in both the nutritional health of these echinoderms and broader ecosystem dynamics [12]. The complex dietary profile of macroalgae, as evidenced in Table 2, underscores the challenge faced in developing formulated feed for gonad enhancement. The wide ranges observed in protein (2.5–44%), carbohydrate (23–75%), and lipid (0.1–4%) content across different macroalgae species reflect the diverse and dynamic nutritional landscape that sea urchins have adapted to over evolutionary time.

This nutritional variability also provides valuable insights into the profile that formulated feed should aim to replicate. Importantly, the proximate analyses of these macroalgae species point towards a medium-protein, high-carbohydrate, and low-lipid profile, aligning with our findings, which will be discussed in subsequent sections.

**Table 2:** Nutritional composition of macroalgae species in the natural diet of various sea urchin species.

Sea Urchin Species	Macroalgae	Type	Protein Range (%)	Carbohydrates Range (%)	Lipid Range (%)	References
*Strongylocentrotus droebachiensis*	*Laminaria* spp.	Kelp	3–21	35.8–60.2	0.5–3.9	[117,118,119,120]
	*Saccharina* spp.	Kelp	3–21	39.3–68.4	0.4–3.6	
*Strongylocentrotus purpuratus*	*Macrocystis pyrifera*	Kelp	3–13.2	47.2–75.3	0.7–1.6	[121,122,123]
	*Nereocystis luetkeana*	Kelp	2.5–15.28	23–54	1.4–4.4	[76,89,124]
	*Saccharina latissima*	Kelp	5.1–21	39.3–68.4	0.2–3.6	[89,117,118,119]
*Heliocidaris erythrogramma*	*Ecklonia radiata*	Kelp	4.1–7.8	58–71.8	0.1–1.7	[125]
	*Undaria pinnatifida*	Kelp	11–24	38.7–53.2	1.4 –3.5	[118]
*Paracentrotus lividus*	*Posidonia oceanica*	Seagrass	4.1–4.8	56–60	2.1–3.2	[126,127]
	*Rissoella verruculosa*	Red	3–12.6	30.5–41.7	0.8–1.7	[127]
*Evechinus chloroticus*	*Ecklonia radiata*	Kelp	4.1–7.8	58–71.8	0.1–1.7	[125]
	*Carpophyllum* spp.	Brown	4–8	36.8–65	1.8–2.5	[128]
	*Ulva* spp.	Green	4–44	38.5–61.5	0.1–2.9	[118,120,129,130]
*Mesocentrotus nudus*	*Saccharina japonica*	Kelp	3–21	39.3–68.4	0.4–3.6	[117,118,119,120,131]
	*Undaria pinnatifida*	Kelp	11–24	38.7–53.2	1.4 –3.5	[118]
*Tripneustes gratilla*	*Sargassum* spp.	Brown	9–20	37.4–49.8	1.2–2.9	[118]
	*Kappaphycus alvarezii*	Red	6.2–6.8	42.1–54.3	0.9–1.0	[132]
*Mesocentrotus franciscanus*	*Macrocystis pyrifera*	Kelp	5.1–12.7	38.6–59.4	0.7–1.1	[121,122,123]
	*Nereocystis luetkeana*	Kelp	2.5–15.3	23–54	1.4–4.4	[76,89,124]
	*Eisenia arborea*	Kelp	5.5–11.7	43.3–54.3	0.45–0.66	[133]

Note that the protein, carbohydrate, and lipid values are approximate and may vary depending on factors such as geographical location, season, and the specific part of the macroalgae analyzed.

### 3.2. Nutritional and Economic Considerations of Formulated Feed for Sea Urchins

The development of formulated feed for sea urchin gonad enhancement presents significant ecological and economic challenges, particularly in sourcing sustainable and cost-effective ingredients (Table 3). Traditionally, the development of aquaculture feed, including sea urchin feed, has relied primarily on fish meal and fish oil as primary sources of protein and essential fatty acids [134,135]. However, these ingredients depend on exploiting wild fish stocks, contributing to overfishing and potentially disrupting marine food chains [136,137]. Moreover, the fishing and processing methods used to produce these ingredients can produce a substantial carbon footprint, contributing to climate change [138,139]. In addition, the high costs associated with the incorporation of fish products into formulated feed impair the economic feasibility of large-scale sea urchin ranching with these ingredients [135]. Fish meal is always subject to price volatility due to the increasing demand from various sectors, including human nutrition and other aquaculture operations [140,141,142]. Finally, fish meal is associated with lower quality taste (bitterness and oiliness) in sea urchin gonads [143,144].

While sustainability and cost concerns have driven exploration of alternatives to fish meal, its nutritional attributes deserve recognition. Fish meal provides highly digestible protein (>60% digestibility), an amino acid profile closely matching aquatic animal requirements (Table 3), and essential omega-3 fatty acids (EPA, DHA) [135]. These attributes explain its historical dominance in aquaculture feeds. The challenge lies not in fish meal’s nutritional inadequacy, but rather in the economic and environmental constraints of scaling its use to meet expanding aquaculture demands globally. Thus, substitution efforts should be viewed as responses to supply and sustainability limitations rather than rejections of fish meal’s nutritional value.

To address these challenges, researchers have explored alternative ingredients, with particular focus on macroalgae and plant-based products. Yet, the nutritional composition and economic considerations of these ingredients play crucial roles in determining their viability for sea urchin feed (Table 3).

Macroalgae are a natural component of the sea urchin diet; therefore, they are often incorporated as a main ingredient of their formulated feed. Brown (kelps *S. japonica* and *U. pinnatifida*), green (*Ulva* spp.), and red (*Palmaria palmata*) macroalgae have been used in feed with good results [17,47,52]. These macroalgae offer unique nutritional profiles with bioactive compounds linked to gonad quality [48]. For instance, kelp species contain high levels of β-carotene (13–30 μg/g), fucoxanthin (0.002–6 μg/g), and lutein (0.01–0.3 μg/g), which are linked to development and high-quality colors in sea urchin gonads [49,85,145,146]. In contrast, these compounds are absent or present in much lower quantities in soybean and fish meal (Table 3), suggesting that even small inclusions of macroalgae in formulated feed could significantly enhance gonad growth and coloration [5,17,47,52,147].

The free amino acids (FAAs) resulting from sea urchins consuming macroalgae are closely associated with the taste of their gonads [4,18,20]. Kaneko et al. [148] categorized the FAAs into three groups: umami-tasting (aspartic acid and glutamic acid), sweet-tasting (alanine, glycine, serine, proline, and threonine), and bitter-tasting (arginine, histidine, isoleucine, leucine, lysine, methionine, phenylalanine, tyrosine, and valine). The FAA composition in gonads is influenced by both the amino acid profile of dietary proteins and the sea urchins’ metabolic processes during protein digestion and amino acid metabolism [4,5]. This relationship between feed protein sources and resulting gonad FAA profiles presents challenges when incorporating different compounds, like fish and soybean meal, into sea urchin formulated feed while maintaining a balance to obtain marketable-quality gonads [5,78,149] (Table 3).

Using macroalgae as main ingredients in formulated feed leads to several hurdles. Large-scale harvesting of wild macroalgae may worsen the current ecological problem and increase diet costs [150,151]. Cultivating macroalgae specifically for feed production could be another option, but premium macroalgae often fetch high prices in human food, cosmetic, and pharmaceutical markets, making their use economically challenging for mass feed production [152] (Table 3). Moreover, the prices of cultivated macroalgae will vary greatly depending on where it is used, making it unviable in regions without large-scale culture.

Given these challenges, plant-based ingredients, particularly soybean products, have emerged as a promising alternative. Soybean products could offer advantages, including lower cost, constant availability, and a relatively high protein content [135] (Table 3). Moreover, soybean meal (400–500 USD/ton) is significantly cheaper than fish meal (1500–2000 USD/ton) and macroalgae (>1000 USD/ton) (Table 3). While soybean products offer potential advantages as alternative protein sources in sea urchin feed, their implementation faces several key challenges that require careful consideration.

The presence of antinutritional factors (ANFs) in soybean products can affect nutrient absorption and sea urchins’ health, limiting its supplementation in formulated feed [135,153]. Specifically, trypsin inhibitors and toxic oligosaccharides can reduce physiological functions and sea urchin survival [154]. Pearce et al. [52] obtained 15% mortality in *S. droebachiensis* fed a diet with 32% macroalgae (*P. palmata* and *Ascophyllum nodosum*) and an inclusion of 27% soybean meal. Similar mortality results have been observed when substituting 30% of fish meal with soy protein concentrate and soybean meal in diets for *Totoaba mcdonaldi* [155] and *Oreochromis niloticus* [61], respectively. These mortalities have been associated with increased enzymatic production in hepatopancreas related to trypsin inhibitors, resulting in inflammation and intestinal inflammation due to the oligosaccharides present in soy products. On the other hand, Cyrus et al. [47] obtained a mortality rate of 7% in *T. gratilla* using a plant-based diet (Maize, wheat bran, lucerne meal, and premium HP 300 soy concentrate without ANFs) with 20% *Ulva* spp. inclusion. In this sense, it is critical to develop new feed additives to improve the absorption and utilization of plant-based nutrients, especially proteins and carbohydrates. Moreover, exploring methods to reduce or neutralize ANFs (probiotics, prebiotics, phytobiotics, and enzymes) in soybean products is key for improving feed efficiency and reducing mortality rates [155,156,157]. Finally, soybean products may present amino acid imbalance, particularly in methionine and lysine, compared to brown macroalgae (Table 3).

Despite these challenges, soybean products represent a more economically viable and environmentally sustainable option for scaling up sea urchin ranching in the short term. Still, it must be carefully balanced to meet nutritional efficacy and health requirements. Future research should focus on optimizing soybean-based formulations that can meet the dietary needs of sea urchins without compromising health, growth rates, and gonad quality.

Alternative protein sources from agricultural byproducts are another option being explored to develop sustainable sea urchin feed. These include preprocessed grain residues, fermented plant proteins, and agricultural processing wastes that could provide cost-effective nutrient sources while supporting circular economy principles [17,20,46]. However, these alternatives must be carefully evaluated for their effects on gonad quality, particularly taste and color, as well as their potential impact on sea urchin health and growth performance.

The economic challenges of macroalgae-based feeds could be partially addressed through innovative sourcing strategies. One potential approach involves utilizing byproducts from human-grade algae processing. These offcuts, which would otherwise be considered waste material, often retain significant nutritional value and beneficial compounds that could enhance gonad quality. For example, sporophylls of *U. pinnatifida* have shown success in enhancing gonad quality in *M. nudus* [17,20]. Additionally, fouling algae, particularly *Ulva* species growing naturally on oyster and mussel farms, represents an untapped resource that could be harvested and incorporated into sea urchin feed formulations. This approach not only provides a sustainable feed source but also creates additional value streams for existing aquaculture operations while potentially reducing their maintenance costs [47].

Nonetheless, these alternatives, while interesting from sustainability and circular economy perspectives, require substantial research and development before they can be implemented at commercial scales. The priority for advancing sea urchin aquaculture remains the development of standardized formulated feeds that balance economic viability with consistent production of high-quality gonads. Such feeds must be readily available, cost-effective, and capable of producing marketable gonads within the 6–12-week culture period that makes sea urchin ranching commercially attractive [17,47,52,79]. While exploration of alternative protein sources should continue, the immediate focus should be on optimizing existing formulations that have demonstrated success in gonad enhancement and quality, perhaps incorporating these alternative ingredients incrementally as their efficacy is proven through rigorous testing.

**Table 3:** Comparative analysis of nutritional composition, bioactive compounds, and estimated costs of key ingredients used in sea urchin formulated feeds.

Costs and Macronutrients	SBM [158,159]	Wakame [160,161]	Rockweed [162,163]	Sea Lettuce [164,165]	Kombu [161,166]	Fish Meal [167]
Costs (USD/ton)	400–500 [168]	2000–4000 [169]	1000–1500 [170]	2000–5000 [169]	3000–6000 [169]	1500–2000 [168]
Macronutrients (%)						
Protein	48	16.3	8.7	15.7	6.2	70
Carbohydrates	34	37	42.6	33.22	35	-
Lipids	0.7	1	2.14	3.8	2	12
Carotenoids (μg/g)						
β-carotene	0.69	13	0.096	0.5	29.9	-
Fucoxanthin	0	0.8	2.52	0.69	1.12	-
Zeaxanthin	0.002	0	0.58	9.47	6	-
Lutein	0.0193	0.015	0.052	10.23	-	-
Amino acids (g/100 g protein DW)						
Phenylalanine + Tyrosine	2.64	12.11	0.5	8.79	3.56	3.8
Methionine + Cysteine	0.67	5.99	0.15	4.32	3.45	3.6
Threonine	1.87	7.33	0.36	6.31	3.41	4.1
Valine	2.45	16.84	0.35	9.15	6.01	4.9
Isoleucine	2.35	7.91	0.29	4.76	2.61	4.3
Leucine	2.69	13.70	0.53	8.25	4.45	7
Lysine	2.69	11.12	0.43	6.50	4.77	7.5
Histidine	1.2	5.25	0.12	1.39	2.38	2.2
Alanine	2.16	27.20	0.65	9.16	4.51	7.7
Arginine	3.74	8.41	0.31	6.19	2.96	5.8
Aspartic acid	5.66	10.18	0.84	12.90	4.69	10
Glutamic acid	9.84	10.65	1.71	12.94	3.86	20.8
Glycine	2.11	8.76	0.41	6.65	3.31	4.1
Tyrosine	1.87	4.31	0.16	5.99	1.74	5.1
Serine	2.5	5.76	0.37	6.94	2.45	4.8

Note: Values presented in this table are representative averages based on the available literature and may vary significantly depending on factors such as geographical origin, season, processing methods, and market conditions. Prices are subject to fluctuation based on supply and demand, transportation costs, and global economic factors. The content of bioactive compounds (e.g., carotenoids, amino acids) can vary substantially based on species, growing conditions, harvesting time, and post-harvest processing. Nutritional composition may also differ depending on the species or the specific part of the ingredient analyzed (e.g., whole algae vs. specific tissues). Readers should consider these values as indicative rather than absolute and are encouraged to consult the most recent and relevant sources for specific applications. The absence of a value does not necessarily indicate that the compound is not present but may reflect a lack of data in the reviewed literature. SBM = soybean meal; DW = dry weight; USD = United States dollars.

## 4. Effect of Formulated Feed on Sea Urchin Gonad Enhancement and Coloration

### 4.1. Literature Review and Data Extraction

To analyze the effects of macronutrients’ proximate composition and feed ingredients on sea urchin gonad growth rate and coloration, we conducted a comprehensive literature review using Web of Science, Scopus, and Google Scholar databases. Our search employed keywords such as “sea urchin,” “gonad enhancement,” “formulated feed,” “gonad production,” “gonad size,” “protein requirement,” “optimum protein,” “dietary protein,” and “gonad color” and specific sea urchin species names, including the former names *Strongylocentrotus franciscanus* and *Strongylocentrotus nudus*. We included studies reporting feed proximal, nutrient profile, and gonad enhancement results using the gonadosomatic index (GI) and color values. We extracted data on protein, carbohydrate, and lipid content. To standardize values between studies, when data was provided with moisture, we converted to dry weight using the following formula:

Dry matter value = (nutrient value in original food / (100 − moisture content)) × 100

We also extracted fish meal, soybean meal, and macroalgae meal percentage when available. Data from 26 experimental treatments across 15 studies were compiled, covering 8 sea urchin species: *S. purpuratus*, *S. droebachiensis*, *E. chloroticus*, *T. gratilla*, *M. franciscanus*, *H. erythrogramma*, *M. nudus*, and *P. lividus*. The studies spanned from 1997 to 2025, with experimental durations ranging from 42 to 120 days or 6 to 17 weeks (Appendix A Table A1 and Table A2).

### 4.2. Data Analysis and Visualization

To standardize comparison across studies with different durations, we calculated the weekly GI increase (WGII, %):

WGII = (GI final − GI initial) / number of weeks

where GI final is the final gonadosomatic index, GI initial is the initial gonadosomatic index, and the number of weeks is the duration of the study converted to weeks. This calculation method allows for a straightforward comparison of gonad growth rates across studies with different lengths and different initial GIs.

For color analysis, we employed a two-pronged approach. When studies provided CIE Lab* color values, we converted these to RGB values and positioned them on a standardized color table https://colordesigner.io/ (accessed on 12 April 2025). We used these directly for studies that provided qualitative color descriptions. We then categorized colors into a qualitative range from best to worst market quality: orange, orange/yellow, yellow, pale orange, pale yellow, and pale yellow/brown. This approach allowed us to integrate quantitative and qualitative color data from diverse studies into a unified analysis framework, facilitating comparisons of gonad color quality across different experiments. Lastly, to explore the relationship between feed composition, nutrient profile, and gonad enhancement outcomes, we developed a series of interactive 3D scatter plots using R (version 4.4.1) with the tidyverse, plotly, and htmlwidgets packages. Compiled data on formulated feed composition and gonad enhancement results for various sea urchin species are available in Appendix A Table A1. Data on feed ingredients and color analysis results are available in Appendix A Table A2.

While our analysis encompasses the available peer-reviewed literature meeting our inclusion criteria, the relatively modest sample size (26 treatments, 15 studies, 8 species) represents a limitation. Additional controlled feeding trials across more species, environmental conditions, and feed formulations would strengthen confidence in the identified optimal composition ranges and ingredient recommendations. Particularly needed are (1) standardized multi-species comparisons using identical feed formulations, (2) studies on additional barren-forming species from underrepresented regions, and (3) longer-term trials evaluating effects beyond single growth cycles. The patterns identified in this review provide a foundation for such expanded research efforts.

### 4.3. Effect of Proximate Macronutrient Composition on Gonad Growth Rate and Color

**Figure 2:**
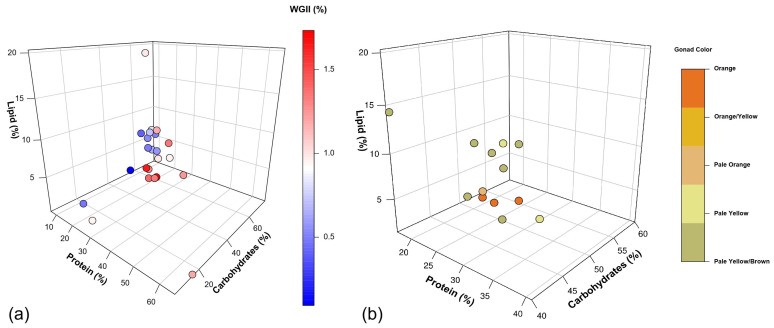
The effect of macronutrients (protein, carbohydrates and lipids) in formulated feed in sea urchin gonad enhancement. (**a**) Weekly gonadosomatic index increase (WGII, %) of sea urchin fed with different proximate compositions (protein, carbohydrates, and lipids, %). The color gradient represents the WGII, ranging from low (blue) to high (red) values. (**b**) Relationship between proximate composition and marketable gonad color. The color of each point represents the resulting gonad color, ranging from orange (best) to pale yellow/brown (worst) quality. The best values used for discussion are from [17,47,52], respectively. For more information, refer to Appendix A, Table A1 and Table A2, where all the data is available.

Our analysis revealed interesting relationships between macronutrient composition and sea urchin gonad growth rate (Figure 2a) and color (Figure 2b). The highest growth rates (1.66–1.73% WGII) and optimal colors were achieved with diets containing 21–28% protein, 48–53% carbohydrates, and 1.2–4% lipids. These findings provide valuable insights into optimal feed formulations for sea urchin gonad enhancement.

### 4.4. Protein Composition

Proteins are fundamental components of all multicellular organisms, playing crucial roles in structure, function, and metabolism [171]. Sea urchins in their natural habitats must consume and digest large quantities of food with relatively low protein content to meet their dietary requirements [172]. This is particularly relevant when developing formulated feed as protein is typically the most expensive macronutrient in aquaculture feed.

Protein content in diets greatly influences gonad growth rates in sea urchins, as it provides energy and essential amino acids for growth and reproduction [43,55,68,134,173]. Interestingly, diets with higher protein content tend to result in lower consumption rates, while low-protein diets stimulate increased feed intake [50,55,134]. This relationship was demonstrated by Hammer et al. [134] in their study with *Lytechinus variegatus*. They found that sea urchins fed a diet containing 20% protein showed superior performance across multiple parameters—including consumption, survival, specific growth rate, and gonad production efficiency—compared to those fed diets with either 9% or 31% protein. Moreover, the 20% protein diet resulted in the highest protein efficiency ratio, indicating optimal protein utilization.

These findings align well with our analysis, which identified highest gonad growth rates at intermediate protein levels of 21 to 28% (Figure 2a). This range appears to reach a balance between providing sufficient protein for growth and avoiding the reduced feed intake associated with high-protein diets. The consistency between our findings and previous studies strengthens the case for optimizing protein levels in sea urchin feed. However, it is important to note that gonad growth is influenced not only by the quantity of dietary protein but also by its source, digestibility, and absorption efficiency [80]. Different protein sources, like fish meal and soybean products, can vary significantly in their amino acid profiles and bioavailability, which in turn affects their nutritional value for sea urchins’ development (Table 3).

Importantly, Takagi et al. [50] suggest that protein content has been overestimated in diets for sea urchins. They evaluated protein leaching in formulated diets after immersion in sea water and concluded that leaching is important even in diets that have not disintegrated. They suggested wheat gluten as a binder due to its viscoelasticity and reported that the optimum level of protein in gluten-based formulated feed for *M. nudus* was 11–13%. This is lower than previous studies, yet as edible sea urchins prefer Laminariales kelp with 4–16% protein, this result seems reasonable and should be further investigated.

The optimization of protein content in sea urchin feed presents an opportunity to balance nutritional efficacy with economic viability. Future research should focus on identifying protein sources that offer the best combination of nutritional value, digestibility, and cost-effectiveness for sea urchin gonad enhancement. Additionally, investigating the potential for protein-sparing effects through the optimization of non-protein energy sources (e.g., carbohydrates and lipids) could further improve feed efficiency and reduce costs.

### 4.5. Carbohydrate Composition

Carbohydrate content in sea urchin diets plays a crucial role in overall nutrition and gonad enhancement. One of its primary functions is the protein-sparing effect, where carbohydrates serve as the main energy source, allowing proteins to be reserved for tissue growth and repair rather than being catabolized for energy [134]. This emphasizes the importance of optimizing carbohydrate absorption to enhance protein utilization and to reduce the reliance on more expensive and environmentally impactful protein sources, such as fish meal [135,155]. Thus, by improving carbohydrate utilization, some other economic and environmental advantages in aquaculture can be obtained [144].

In natural diets, carbohydrates range from 23 to 75.3% (Table 2), suggesting that sea urchins are adapted to utilize varying levels of carbohydrates efficiently. Our analysis suggests that high carbohydrates (48–53%), combined with medium protein (21–28%) and low lipids (1.2–4%), produced the highest gonad growth rates (Figure 2a) and colors (Figure 2b). Interestingly, the carbohydrate levels in potential feed ingredients like soybean meal and various macroalgae species (33–43%, Table 3) align well. This suggests that blending these ingredients could achieve the desired carbohydrate levels while improving overall nutrient profiles [162].

The relationship between carbohydrates and gonad quality extends beyond growth rates. Pearce et al. [144] suggested that the balance of macronutrients, including carbohydrates, can significantly affect the taste of *S. droebachiensis* gonads. They found that diets with high protein levels were associated with bitter-tasting gonads and that this could be mitigated by adjusting the carbohydrate content, as glycogen and its breakdown products may contribute to the sweet taste of high-quality sea urchin gonads. However, an excessive level of carbohydrate in diets can cause undesirable changes in the composition and taste of the gonad, highlighting the importance of balancing macronutrients for gonad growth rates and quality while maintaining economic and ecological sustainability.

Using plant ingredients in formulated feed for sea urchin has challenges related to the cellulose content, as sea urchins have limited expression of cellulase genes [174] and cellulose activity [175]. Future research should focus on (1) optimizing carbohydrate types and levels in formulated feed to maximize the protein-sparing effect without compromising gonad quality; (2) investigating the role of different carbohydrate sources (e.g., from various macroalgae or plant-based ingredients) on gonad quality; (3) exploring the potential of carbohydrate-degrading enzymes or probiotics to enhance carbohydrate utilization in sea urchins, particularly in soybean products that contain less digestible complex carbohydrates; and (4) examining the interaction between carbohydrates and other feed components, such as pigments, which may influence gonad coloration and market value.

### 4.6. Lipid Composition

The role of lipids in sea urchin nutrition and gonad development is complex. Our analysis revealed a threshold for lipid content in formulated feed, with growth rates and color declining when dietary lipid content exceeded 4% (Figure 2a,b). This finding aligns with the natural diet of sea urchins, which typically contains low lipid levels (0.1–4%) (Table 2). Our results are in accordance with those of Suckling et al. [146] and Gibbs et al. [176], who documented that an increment in lipid levels from 2.2% to 8.4% and 1% to 8.8%, respectively, resulted in reduced gonad growth rates. Additionally, Warren-Myers et al. [56] also obtained lower gonad growth rates in *H. erythrogramma* when fed high (131 g of lipids per kg of feed) and medium (101 g/kg), compared to low (73 g/kg), lipid levels.

This decline in gonad growth rate and color with higher level of lipids could be attributed to several factors: (1) sea urchins’ limited capacity to metabolize high levels of dietary lipids [177]; (2) lipids potentially leading to reduced feed intake or impaired nutrient absorption [178]; (3) high lipid levels potentially disrupting the balance of other essential nutrients, potentially leading to suboptimal nutrient utilization [179]; and (4) lipid oxidation in high-fat diets potentially producing harmful byproducts, potentially causing metabolic stress that could reduce gonad growth rate [180].

Angwin et al. [17] found that despite varying dietary lipid levels from 0.04% to 1.24%, the final gonad lipid composition was between 3.5 to 3.9% in *S. purpuratus*. Similarly, Liyana-Pathirana et al. [71] reported that *S. droebachiensis***,** whether fed on macroalgae-rich diets or living in the wild, typically had gonad lipid compositions of approximately 3.5–4%. This consistency in lipids concentration suggests tightly regulated lipid homeostasis in sea urchin gonad development [181]. In this sense, our results support these findings, indicating that dietary lipid levels exceeding 4% may affect gonad growth rate (Figure 2a) and color (Figure 2b). These results are in agreement with González-Durán et al. [177], who found that while dietary lipids influenced fatty acid profiles in sea urchin tissues, there are physiological limits to lipid accumulation, particularly in gonads. They noted that excessive dietary lipids could lead to reduced feed intake or impaired nutrient absorption.

The low optimal lipid content for sea urchin feed (1.5–4%) contrasts sharply with the high lipid content of fish meal (~12%, Table 3), which has been used as a major component of sea urchin feed. This discrepancy underscores the need to reconsider the use of fish meal in sea urchin feed, not only for ecological and economic reasons but also for nutritional optimization. Interestingly, both plant-based ingredients like soybean meal and various macroalgae species have lower lipid content (0.7–3.8%, Table 3) compared to fish meal, aligning more closely with the optimal lipid levels for sea urchin gonad enhancement, further supporting the potential of plant-based and macroalgae-derived ingredients in formulated sea urchin feed.

## 5. Effect of Feed Ingredients on Gonad Growth Rate and Color

Feed ingredients play a crucial role in sea urchin gonad enhancement, influencing both growth rates and marketable color qualities. Our analysis reveals interesting differences in performance between macroalgae-based, plant-based, and fish meal-based diets.

### 5.1. Macroalgae- and Plant-Based Diets

**Figure 3:**
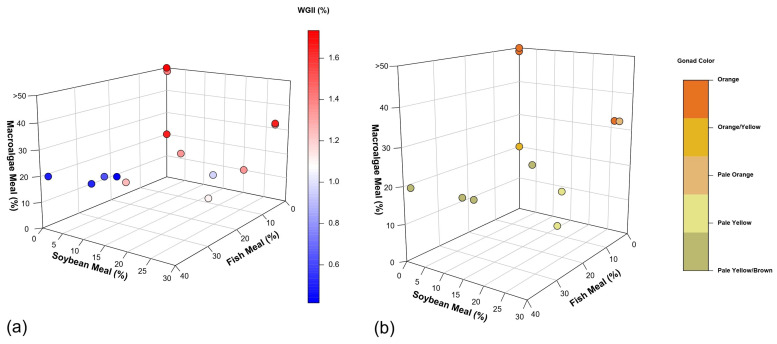
(**a**) Weekly gonadosomatic index increase (WGII, %) relationship between fish meal (%), soybean meal (%), and macroalgae meal (%) in diets. The color gradient represents the WGII, ranging from low (blue) to high (red) values. (**b**) Relationship with marketable gonad colors. The color of each point represents the resulting gonad color, ranging from orange (best quality) to pale yellow/brown (worst quality). The best values used for discussion are from [17,47,52], respectively. For more information, refer to Appendix A, Table A1 and Table A2, where all the data is available.

The highest growth rates (1.66–1.73%) in WGII (Figure 3a) and marketable color qualities (Figure 3b) were observed in diets containing more than 20% of macroalgae [17,47,52], aligning with evolutionary adaptation of sea urchins to efficiently assimilate nutrients from macroalgae [12]. The peak growth rate (1.73% WGII) was achieved with a diet primarily composed of human-grade Kombu, *S. japonica* [17]. While the exact formulation is proprietary, the absence of fish meal and soybean products suggests a high macroalgae content. However, the high costs associated with human-grade macroalgae could hinder large-scale production and industry scalability.

Pearce et al. [52] reported the second-highest growth rate (1.67% WGII) using a diet containing 32% macroalgae (*Ascophyllum nodosum* and *P. palmata*) and 27% soybean meal. This formulation represents a significant step towards cost reduction through the partial substitution of expensive macroalgae with more affordable plant protein. However, it faces challenges including ecological concerns related to wild harvesting of *A. nodosum* [182] and potential cost increases due to the growing popularity of *P. palmata* for human and animal consumption [183,184].

Cyrus et al. [47] also achieved a 1.67% weekly growth rate using plant-based feed (maize, wheat bran, soy HP 300 protein, and lucerne meal) with 20% *Ulva* spp. inclusion. While this formulation achieved the same growth rate with a lower mortality rate (7%) than the formulation used by Pearce et al. [52,155], who obtained a 15% mortality rate using 27% soybean meal, it still faces economic challenges due to the cost of cultivated *Ulva* spp. and premium HP 300 soy protein without ANFs. This approach, however, demonstrates the potential for diversifying plant-based ingredients and exploring methods to reduce or neutralize ANFs to enhance overall performance in sea urchin feed.

### 5.2. Fish Meal

**Figure 4:**
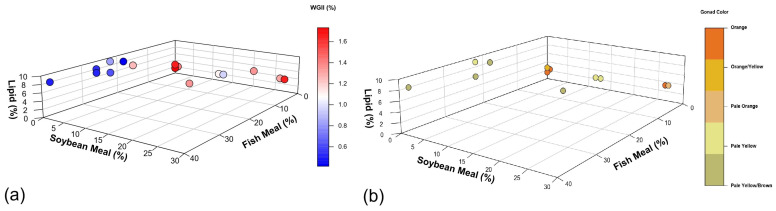
(**a**) Weekly gonadosomatic index increase (WGII, %) relationship between fish meal (%), soybean meal (%), and lipids (%) in formulated feed. The color gradient represents the WGII, ranging from low (blue) to high (red) values. (**b**) Relationship with marketable gonad colors. The color of each point represents the resulting gonad color, ranging from orange (best quality) to pale yellow/brown (worst quality). The best values used for discussion are from [17,47,52], respectively. For more information, refer to Appendix A, Table A1 and Table A2, where all the data is available.

Fish meal exceeding 13% in sea urchin feed appears to have adverse effects on gonad growth rate (Figure 3a and Figure 4a) and gonad coloration (Figure 3b and Figure 4b). This negative impact may be attributed to the high levels of animal-derived nitrogen in fish meal. Olmos et al. [155] found that excessive levels of fish meal in aquaculture diets can disrupt nitrogen homeostasis in the cultivated species. Additionally, high levels of fish meal can lead to increased ammonia excretion, potentially causing water quality issues in aquaculture systems. In the case of sea urchins, this excess of nitrogen and poor water quality could potentially interfere with their metabolic processes, leading to reduced gonad growth rate and color quality. In addition, the high lipid content in fish meal (~12%) limits its inclusion in sea urchin feed compared to macroalgae- and plant-based alternatives (0.1–4%; Table 3).

Contrary to 13% fish meal, 27% inclusion of soybean meal does not appear to have a negative effect on growth and color (Figure 4a,b), suggesting promise for plant-based diets [52]. However, Liyana-Pathirana, Shahidi, and Whittick [71] and Woods et al. [103] found that despite a well-balanced proximate composition in a plant-based diet, the resulting gonad protein content was only 8% in *S. droebachiensis* and *E. chloroticus*, respectively. These results highlight the need for further research to enhance plant-based protein assimilation efficiency in sea urchin diets to reach the 12% found in wild sea urchin gonads [17,185].

Further incorporation of plant-based ingredients aligns with the aquaculture industry’s trend towards reducing animal-derived protein use due to sustainability concerns and rising costs [141]. However, future studies must optimize blends of macroalgae and soybean to balance performance with cost-effectiveness and sustainability. Moreover, it is crucial to investigate methods to improve digestibility and nutritional value of plant-based proteins for sea urchins, especially soybean [61,62,63,155]. Finally, we should adopt a holistic approach, considering not only growth rates and coloration but also gonad taste, texture, and market acceptability balanced with feed costs and sustainability [2].

### 5.3. Effect of Feed Ingredients on Gonad Coloration

Gonad coloration in sea urchins is fundamentally determined by the accumulation and metabolic transformation of dietary carotenoids, with market value directly correlating to pigmentation intensity and hue. Since sea urchins cannot synthesize carotenoids de novo, gonadal accumulation depends exclusively on dietary carotenoid concentration and bioavailability. The predominant gonadal carotenoid is 9′-cis-echinenone, accounting for 90–100% of total carotenoids in high-quality, orange-colored gonads, produced through enzymatic transformation of dietary β-carotene. Echinenone is synthesized via β-carotene oxidation in the gut, then transported by low-density lipoproteins to the gonads [3,113]. This lipid-dependent transport explains the observed relationship between dietary lipid levels (1.2–4%) and optimal gonad coloration (Figure 2b), as insufficient lipids impair carotenoid absorption while excessive lipids may cause metabolic imbalances.

### 5.4. Metabolic Pathways and Compartmentalization

Nutritive phagocytes (NPs) within gonadal tissue mediate carotenoid accumulation and storage through a three-step pathway: (1) dietary β-carotene undergoes enzymatic oxidation by β-carotene ketolase in the gut, introducing a keto group at the C-4 position to form echinenone (4-keto-β-carotene); (2) all-trans-echinenone isomerizes to 9′-cis-echinenone, the thermodynamically stable configuration favored in the lipid-rich environment; and (3) 9′-cis-echinenone is transported in lipoprotein droplets and deposited in NP, creating the characteristic orange coloration valued commercially. Comparative studies across multiple species demonstrate that β-echinenone is the major gonadal carotenoid in 19 of the 20 examined species [186,187,188]. Individuals fed β-echinenone-supplemented diets showed five-fold higher echinenone concentrations versus controls, indicating efficient metabolite accumulation [186,187].

### 5.5. Alternative Carotenoid Pathways and Biological Functions

While α-carotene, lutein, astaxanthin, fucoxanthin, and other xanthophylls accumulate at minor concentrations in gonads [71,187,189,190,191], they may serve as protective antioxidant sources. Lutein absorption and retention appear less efficient than β-carotene absorption and retention, possibly due to its more polar structure, though it may contribute to yellow gonad tones and antioxidant protection [192].

Although fucoxanthin, the major carotenoid in brown algae (*Laminaria* spp., *U. pinnatifida*, *E. bicyclis*) constituting natural sea urchin diets [160,166], and its metabolite fucoxanthinol were detected in *P. depressus* viscera, neither accumulated in the gonads, suggesting that sea urchins lack mechanisms to incorporate these xanthophylls into gonadal tissue [188]. Despite absent gonadal accumulation, fucoxanthin significantly enhanced phagocytic activities (three-fold higher phagocytic index versus controls) and egg ovulation numbers. Remarkably, ovulated egg numbers exceeded those in β-carotene-fed groups, suggesting that fucoxanthin exerts beneficial effects during critical reproductive stages [188].

Astaxanthin presents complex, species-specific accumulation patterns. While *P. depressus* accumulated minimal astaxanthin from supplemented diets [188], *Arbacia lixula* demonstrated remarkable accumulation capacity in eggs when fed astaxanthin precursor-containing diets. Farmed *A. lixula* showed egg astaxanthin concentrations of 27.0 μg/mg, fifteen-fold higher than wild specimens (1.5 μg/mg), with astaxanthin as the sole detected pigment [193]. Total astaxanthin reached approximately 2.7% of egg dry weight in cultivated individuals [193], suggesting variable metabolic capabilities among species regarding xanthophyll biotransformation.

### 5.6. Proposed Mechanisms for Carotenoid Effects on Gonad Development and Quality

Carotenoids’ influence on gonad development extends beyond pigmentation to encompass critical biological defense functions. Carotenoids, particularly β-carotene and echinenone, significantly enhance biological defense mechanisms, including phagocytic activities [188]. Echinenone demonstrated the strongest phagocytic enhancement, with a phagocytic index 7.4-fold higher than controls, surpassing even vitamin A and vitamin E [188]. Phagocytic ratios increased 2.5-fold for echinenone and 2.1-fold for β-carotene versus controls [188].

This immunoenhancement likely occurs through protection of phagocytic cells from autooxidative damage by quenching singlet oxygen and reactive oxygen species generated during phagocytosis [194,195]. Sea urchin phagocytic cells produce reactive oxygen species when engulfing foreign materials [196], and while these products aid bacterial killing, they can damage DNA and cell membranes. Carotenoids protect phagocytic cells from this autooxidative damage through antioxidant properties [194,195].

Carotenoid deficiency consequences were demonstrated through disease characterized by spine loss and underdeveloped gonads in sea urchins fed carotenoid-free diets, with mortality reaching 30% in control groups versus zero in carotenoid-supplemented groups [188]. These symptoms were attributed to decreased biological defense from carotenoid deficiency [188].

Feeding trials revealed that gonads increased significantly in size regardless of diet type, indicating that carotenoids at the tested concentrations did not directly influence gonad growth [187]. However, absolute carotenoid amounts in gonad tissue increased significantly over time despite decreasing concentrations, suggesting either that gonad growth exceeded carotenoid deposition rates or dietary carotenoid amounts were suboptimal [187].

### 5.7. Applications in Formulated Feed Development

While formulated feeds effectively promote gonadal development, achieving optimal color quality remains challenging when combining algae and plant meals [194]. To address this, researchers have evaluated natural and synthetic β-carotene inclusion in feed formulations [49,85,101,103,146,187]. Robinson et al. [49] demonstrated that 250 mg β-carotene kg^−1^ dry feed produces desirable *S. droebachiensis* gonad coloration. However, the high costs and estimated 50–75% pigment degradation during pellet manufacture and storage have limited commercial production [146,191,197,198,199,200], prompting exploration of dried or fresh macro- and microalgae as more cost-effective, stable natural carotenoid sources.

Macro- and microalgae inclusion has yielded promising results for gonad growth rates (Figure 3a), palatability, and coloration (Figure 3b) [17,47,52,54]. McLaughlin and Kelly [201] demonstrated that 70% *Phaeodactylum* microalgae incorporation in wet diets significantly improved *P. miliaris* gonad coloration. Our analysis indicates that >20% macroalgae inclusion positively affects gonad coloration in multiple species, including *S. droebachiensis* [52], *S. purpuratus* [17], and *T. gratilla* [47] (Figure 3b). However, these findings require cautious interpretation due to metabolism, temperature, light exposure, season [56,71], diet ingredients [187], maturation stage [189], and sex effects on gonad coloration [113,190,202].

Temperature is the most influential environmental factor affecting sea urchin metabolism, feed consumption, and gonad development rates. Each species exhibits an optimal temperature range for growth and gonad production, with performance declining outside these bounds. For example, *S. droebachiensis* shows maximal feed consumption at 12–14 °C, with consumption declining at both lower (6–8 °C) and higher (16–18 °C) temperatures [70]. Also, *S. purpuratus* demonstrates optimal feeding activity at 16 °C, with reduced consumption below 12 °C [89]. Additionally, *M. nudus* exhibits temperature-dependent feeding, with peak consumption at 15 °C, declining with lower (10 °C) or higher (20 °C) temperatures [36]. These temperature–consumption relationships create a fundamental challenge: at temperatures below optima, sea urchins consume insufficient feed to support rapid gonad growth, while at temperatures above optima, consumption may decline due to metabolic stress despite elevated metabolic demands.

Temperature affects not only the quantity of feed consumed but also the efficiency of nutrient utilization. Optimal protein retention efficiency typically occurs at mid-range temperatures within species tolerance. At low temperatures, reduced metabolic rates may improve feed conversion ratios but slow absolute growth rates. At high temperatures, increased maintenance energy requirements reduce the proportion of nutrients allocated to gonad production [203]. Additionally, digestive enzyme production and activity show temperature-dependent patterns, potentially affecting the digestibility of carbohydrates which are crucial for thermoregulation and movement [78]. Moreover, higher temperatures generally increase lipid catabolism for energy [204], potentially reducing lipid deposition in gonads. Plant-based ingredients with lower inherent digestibility may be particularly sensitive to temperature effects. This temperature sensitivity has direct implications for commercial ambient water operations that must account for seasonal variation in growth rates and the relation with feed efficiency.

Sex-related coloration differences have been well-documented, with females generally exhibiting more attractive marketable colors due to differential nutrient and pigment allocation to gonads [200,202]. Hagen et al. [202] suggested that synthetic carotenoid supplementation may be inefficient for male *S. droebachiensis*, which naturally produce paler gonads than females. Lourenço et al. [200] evaluated the combined effects of sex, fishmeal, macroalgae, plant-based ingredients, and 100 mg kg^−1^ β-carotene on *P. lividus* gonad coloration, finding that (1) carotenoid source and concentration were insufficient to promote gonadal pigment accumulation, potentially due to feed formulation degradation; (2) carotenoid content decreased with increasing gonad weight; and (3) sex significantly influenced both color development and carotenoid content, with females consistently achieving superior results.

The environmental variability discussed above—combined with species-specific physiological differences, regional variations in natural diet composition, and seasonal fluctuations in nutritional demands— introduces heterogeneity into our data synthesis. While our approach of standardizing growth rates using the WGII partially controls for different culture durations, it cannot eliminate the confounding effects of temperature, photoperiod, salinity, or developmental stage variations across studies. Consequently, the optimal macronutrient composition ranges identified in our analysis (21–28% protein, 48–53% carbohydrates, 1.2–4% lipids) should be interpreted as robust general trends emerging across diverse experimental conditions rather than precise prescriptions applicable to all species, regions, and seasons. The patterns we identify represent central tendencies that persist despite environmental heterogeneity, suggesting fundamental nutritional requirements common across barren-forming sea urchins. However, practitioners implementing these findings should recognize that finetuning feed formulations for specific species, geographic locations, seasonal conditions, and target market specifications will likely require region-specific optimization trials. Our results provide a scientifically grounded starting framework for such optimization efforts rather than universal optima applicable without local adaptation.

### 5.8. Integrated Mechanistic Model and Future Directions

Developing sustainable feeds for optimal gonad growth and coloration may benefit from plant-based ingredients, particularly soybean meal, which contains notably higher carotenoid levels than fish meal (Table 3). Functional feed formulations including probiotics, prebiotics, phytobiotics, and specialized enzymes present promising approaches to enhance plant-based nutrient assimilation and carotenoid metabolic conversion into gonad pigmentation, potentially reducing reliance on expensive macro- and microalgae while maintaining high-quality gonad coloration standards.

Based on current evidence, we propose an integrated mechanistic model for carotenoid effects on sea urchin gonad development and quality, encompassing four interconnected pathways.

First, the digestive bioconversion pathway establishes that β-carotene from plant-based ingredients undergoes enzymatic ketolation by β-carotene ketolase in the gut wall to form echinenone before gonadal transport [186,187,188], with nutritive phagocytes in gonads functioning primarily in storage and accumulation.

Second, the lipoprotein transport mechanism conveys echinenone through coelomic fluid via low-density lipoproteins to gonadal tissue [3,113], explaining the critical relationship between dietary lipid levels (1.2–4%) and optimal carotenoid accumulation.

Third, immunological enhancement and biological defense mechanisms show that carotenoids, particularly echinenone, protect developing gametes and somatic tissues through enhanced phagocytic defense (7.4-fold increased phagocytic index) and antioxidant activity quenching reactive oxygen species during immune responses [188,193,194,196], explaining spine loss disease and underdeveloped gonads in carotenoid-deficient sea urchins.

Fourth, alternative xanthophyll pathways reveal that carotenoid benefits may operate through systemic effects on maternal physiology and immunity, not solely through gonadal pigment accumulation. Fucoxanthin and other xanthophylls from brown algae, while not accumulating in gonads, enhance reproduction (increased egg ovulation exceeding β-carotene-fed groups) and immunity through metabolic action in digestive tissues or via undetected metabolites [188]. Astaxanthin demonstrates species-specific accumulation patterns, with some species like *A. lixula* achieving high-level egg accumulation (2.7% dry weight) while others show minimal incorporation [188,193], differences that may reflect phylogenetic constraints, ecological adaptations, or life-history correlates requiring systematic comparative investigation.

This approach offers multiple sustainability advantages: reducing feed costs through premium algal ingredient substitution, decreasing dependence on environmentally problematic fish meal, and alleviating wild macroalgae overharvesting. Future research should prioritize optimizing synergistic combinations of plant-based ingredients and functional additives to develop economically viable feed formulations consistently producing market-color-quality gonads while supporting long-term sea urchin aquaculture sustainability.

Based on our comprehensive review of sea urchin gonad enhancement through formulated feed, several key areas for future research and development emerge. The economic feasibility and scalability of formulated feed in commercial settings require thorough trials, alongside efforts to develop cost-effective alternatives to premium ingredients while maintaining optimal gonad quality. Optimizing soybean-based feed remains a priority, alongside investigating methods to reduce or neutralize ANFs and exploring optimal inclusion levels that balance growth performance, gonad quality, and cost-effectiveness [58,59,60,61,62,63]. Developing and testing feed formulations that combine soybean products with strategic inclusions of macroalgae could leverage their unique nutritional properties, such as carotenoids for gonad coloration.

Ecological impact studies should document the broader effects of sea urchin ranching, emphasizing its potential for restoring kelp forests and enhancing biodiversity in urchin barren areas. Long-term studies are necessary to understand the environmental implications of restorative aquaculture practices fully. The potential of Integrated multitrophic aquaculture (IMTA) systems, incorporating sea urchins with complementary species such as seaweed and sea cucumbers, warrants exploration to maximize resource use, minimize environmental impact, and potentially improve sea urchin gonad quality.

Finally, collaboration with policymakers is crucial to develop regulations that support sustainable sea urchin ranching practices while protecting wild populations and ecosystems. Investigating potential incentives or support systems for sea urchin ranchers adopting sustainable, plant-based feed formulations could accelerate the industry’s transition towards more environmentally friendly practices. These multifaceted research directions aim to address the complex challenges in sea urchin aquaculture, balancing economic viability with ecological sustainability and product quality.

## 6. Conclusions

This review provides a critical framework in optimizing formulated feed for sea urchin gonad enhancement, with implications for both aquaculture development and ecosystem restoration. The results obtained reveal that the best feed compositions balance is moderate protein (21–28%), high carbohydrate (48–53%), and low lipid (1.2–4%) levels, with a preference for macroalgae- and plant-based sources.

Macroalgae-based feed demonstrated superior performance in terms of gonad growth rates and marketable colors. However, their economic and ecological viability on a commercial scale remains a challenge, particularly when using premium ingredients. Therefore, for advancing the sea urchin ranching industry, we need innovative feed formulations that strike a balance between performance, gonad quality, cost-effectiveness, sustainability, and scalability.

Our findings suggest that partial substitution of macroalgae with soybean meal could be a solution to reduce feed costs and ecological impacts while maintaining adequate growth and gonad development. However, further research is crucial to determine optimal inclusion levels, as well as the development of biotechnological alternatives (probiotics, prebiotics, phytobiotics, and enzymes) to mitigate antinutritional factors and avoid health problems. As the sea urchin ranching industry evolves, refinement of feed formulations based on plant ingredients will be crucial. This approach has the potential to transform sea urchin ranching into a viable tool for restorative aquaculture, contributing to both the blue economy and ecosystem preservation.

## Data Availability

All data needed is available in the study.

## Abbreviations

The following abbreviations are used in this manuscript:

GI	Gonadosomatic index
WGII	Weekly gonadosomatic index increase
ERP	Ecological restoration potential
FAA	Free amino acids
FF	Formulated feed
ANFs	Anti-nutritional factors
DW	Dry weight
IMTA	Integrated multi-trophic aquaculture

## Appendix A

### Appendix A.1

**Table A1:** Formulated feed composition, experimental conditions, and gonad enhancement results for various sea urchin species. Proximate composition of the diets (protein, carbohydrates, and lipids in dry weight %), time of the studies, initial size, weight and gonadosomatic indexes (GI) from the different sea urchin species and studies. Pro = protein; Carb = carbohydrates; Lip = lipids; D = days; W = weeks; IGI = initial GI; FGI = final GI; TGII = total GI increase; WGII = weekly GI increase.

Pro (%)	Carb (%)	Lip (%)	D	W	Size (mm)	Weight (g)	IGI (%)	FGI (%)	TGII (%)	WGII (%)	Sea Urchin Species	References
21	53	1.2	63	9	52.5	54	6.8	19.56	187.6471	1.417778	*Strongylocentrotus purpuratus*	[17]
24	55	1.5	63	9	52.5	54	6.8	22.4	229.4118	1.733333	*Strongylocentrotus purpuratus*	[17]
25	54	9	84	12	58.2	82.4	9.7	24	147.4227	1.191667	*Strongylocentrotus purpuratus*	[89]
40.8	26.2	20	56	8	75.6	174.8	4.3	12.3	186.0465	1	*Evechinus choloritus*	[103]
24.5	49.6	9	56	8	75.6	174.8	4.3	10.1	134.8837	0.725	*Evechinus choloritus*	[103]
65.2	16	0.3	56	8	54.3	68.1	8.3	17.9	115.6627	1.2	*Strongylocentrotus droebachiensis*	[205]
28	48	2.6	63	9	60	99	7	22	214.2857	1.666667	*Tripneutes gratilla*	[47]
28	48	2.6	84	12	NP	177	6	22.2	270	1.35	*Tripneutes gratilla*	[79]
23	14	0.1	120	17	NP	248	3.4	20	488.2353	0.968333	*Mesocentrotus franciscanus*	[76]
22	50	3.1	42	6	55.8	74.2	4.9	12.7	159.1837	1.3	*Strongylocentrotus droebachiensis*	[52]
22	50	3.3	42	6	45.8	42.2	4	14	250	1.666667	*Strongylocentrotus droebachiensis*	[52]
39	43	7.3	84	12	44	46.3	2.7	14.9	451.8519	1.016667	*Heliocidaris erythogramma*	[111]
39	43	7.3	84	12	47	53.7	2	15.3	665	1.108333	*Heliocidaris erythogramma*	[111]
39	43	7.3	84	12	44	46.3	2.7	15.9	488.8889	1.1	*Heliocidaris erythogramma*	[111]
39	43	7.3	84	12	47	53.7	2	13.6	580	0.966667	*Heliocidaris erythogramma*	[111]
25	55	4.6	84	12	40.18	29.62	6	18	200	1	*Paracentrotus lividus*	[85]
16	77	4.3	63	9	33	43.7	9.1	21.3	134.0659	1.355556	*Strongylocentrotus droebachiensis*	[71]
28	6.3	4.3	84	12	45.73	39.52	13.32	19.16	43.84384	1.46	*Paracentrotus lividus*	[84]
23	53	9	84	12	NP	53.9	9.3	19.3	107.5269	0.833333	*Strongylocentrotus purpuratus*	[206]
23	53	6	84	12	NP	53.9	9.3	17	106	0.64	*Strongylocentrotus purpuratus*	[206]
30	47	7	63	9	NP	35.9	3.3	8.5	157.5758	0.577778	*Strongylocentrotus purpuratus*	[207]
23	50	8	63	9	NP	35.9	3.3	8.2	169.697	0.544444	*Strongylocentrotus purpuratus*	[207]
17	64	7	63	9	NP	35.9	3.3	8	142.4242	0.522222	*Strongylocentrotus purpuratus*	[207]
30	41	8	84	12	NP	45	8.7	14.7	68.96552	0.5	*Strongylocentrotus purpuratus*	[208]
17	53	8	84	12	NP	45	8.7	13.7	57.47126	0.416667	*Strongylocentrotus purpuratus*	[208]
47	40	6.17	84	12	NP	53.2	2.9	18.5	537.931	1.3	*Heliocidaris erythogramma*	[56]
47	40	6.17	84	12	NP	58.2	6.6	21.7	228.7879	1.258333	*Heliocidaris erythogramma*	[56]
6.45	56.74	0.49	84	12	49.8	54.6	8.1	9.2	13.58025	0.091667	*Mesocentrotus nudus*	[5]

### Appendix A.2

**Table A2:** Color analysis, feed composition, and ingredient inclusion levels in sea urchin gonad enhancement studies. Values of Lab, converted RGB, color range obtained, and the amount of fish meal (FM, %), soybean meal (SBM, %), and macroalgae (M, %) in different diets per study. NP = not presented.

Sea Urchin Species	Lab	RGB	Color	FM (%)	SBM (%)	M (%)	References
*Strongylocentrotus purpuratus*	NP	NP	Orange	0	0	>50	[17]
*Strongylocentrotus purpuratus*	NP	NP	Orange	0	0	>50	[17]
*Strongylocentrotus purpuratus*	50.1, 7, 31	rgb (147, 114, 66)	Pale Yellow/Brown	NP	NP	NP	[89]
*Evechinus choloritus*	51.4, 12.9, 28	rgb (158, 113, 75)	Pale Yellow/Brown	NP	NP	NP	[103]
*Evechinus choloritus*	51.7, 12.2, 25.1	rgb (156, 115, 81)	Pale Yellow/Brown	NP	NP	NP	[103]
*Strongylocentrotus droebachiensis*	NP	NP	NP	89.05	0	8.91	[205]
*Tripneutes gratilla*	62, 14, 34	rgb (191, 139, 90)	Orange/Yellow	0	0	20	[47]
*Tripneutes gratilla*	56, 7, 30	rgb (162, 129, 82)	Pale Yellow/Brown	12	12	20	[79]
*Strongylocentrotus droebachiensis*	41, 24, 34	rgb (145, 80, 41)	Orange	0	27	32	[52]
*Heliocidaris erythogramma*	NP	NP	Pale Yellow	14	20	5	[111]
*Heliocidaris erythogramma*	NP	rgb (208, 206, 174)	Pale Yellow	14	20	5	[111]
*Heliocidaris erythogramma*	NP	rgb (208, 206, 174)	Pale Yellow	14	21	15	[111]
*Strongylocentrotus droebachiensis*	NP	NP	NP	0	20	10	[71]
*Strongylocentrotus purpuratus*	60, 15.8, 37.9	rgb (189, 133, 78)	Pale Yellow	22	0	11	[206]
*Strongylocentrotus purpuratus*	57, 15.4, 34.8	rgb (179, 125, 76)	Pale Yellow/Brown	22	0	11	[206]
*Strongylocentrotus purpuratus*	NP	NP	NP	26	0	10	[207]
*Strongylocentrotus purpuratus*	NP	NP	NP	26	0	10	[207]
*Strongylocentrotus purpuratus*	NP	NP	NP	26	0	10	[207]
*Strongylocentrotus purpuratus*	51, 14, 34	rgb (160, 111, 63)	Pale Yellow/Brown	38	0	19	[208]
*Strongylocentrotus purpuratus*	49, 14, 32	rgb (154, 106, 62)	Pale Yellow/Brown	18	0	9	[208]
*Heliocidaris erythogramma*	NP	NP	NP	15	0	5	[56]
*Heliocidaris erythogramma*	NP	NP	NP	15	0	5	[56]
